# Clinical and Imaging Outcomes of the Upside‑Down Pars Plana Ahmed Glaucoma Valve Implantation: A Follow‑Up to Our Initial Report

**DOI:** 10.7759/cureus.101285

**Published:** 2026-01-11

**Authors:** Aya Takiguchi, Yoichiro Masuda, Yuka Saito, Tadashi Nakano

**Affiliations:** 1 Department of Ophthalmology, Jikei University School of Medicine, Tokyo, JPN

**Keywords:** ahmed glaucoma valve, glaucoma drainage device, neovascular glaucoma, refractory glaucoma, tube shunt surgery

## Abstract

Introduction: Tube shunts are essential for refractory glaucoma, yet plate encapsulation can compromise long-term efficacy. We evaluated long-term clinical and imaging outcomes of an “upside-down” Ahmed glaucoma valve (AGV) technique via the pars plana, which may favorably modify bleb architecture.

Methods: This retrospective observational study included 10 eyes from 10 patients who underwent pars plana AGV implantation with the valve plate flipped upside down to direct the drainage port toward the scleral surface. Outcomes included intraocular pressure (IOP), glaucoma medication score, postoperative complications, and corneal endothelial cell density (ECD). Orbital magnetic resonance imaging (MRI) was performed in two eyes to assess bleb morphology.

Results: Mean follow-up was 45.9 ± 24.2 months. Mean preoperative IOP decreased from 35.8 ± 4.4 to 14.9 ± 0.6 mmHg at 66 months (p < 0.05). The glaucoma medication score decreased from 4.6 ± 0.7 to 2.4 ± 0.5 at 42 months (p < 0.05). Transient postoperative hypertension occurred in seven eyes and resolved without surgical intervention. Three patients lost light perception due to retinal ischemia related to the underlying disease. No persistent hypotony or tube exposure was observed. Orbital MRI obtained at approximately six years postoperatively demonstrated preservation of a double-layered peri-endplate bleb configuration in two eyes. The average monthly ECD loss rate was 0.28% ± 0.26%.

Conclusions: The upside-down pars plana AGV technique achieved sustained IOP reduction through 66 months and suggested stable long-term bleb morphology on MRI without adjunctive antifibrotic agents. This approach may be a useful option for complex refractory glaucoma.

## Introduction

Pars plana Ahmed glaucoma valve (AGV) implantation is a well-established approach for managing refractory glaucoma, particularly in eyes with compromised anterior segments or when anterior chamber tube placement poses a higher risk to the corneal endothelium [1,2]. However, long-term outcomes can be limited by postoperative complications, including fibroproliferative responses and encapsulation around the endplate [3,4].

In 2021, we introduced an “upside-down” technique in which the AGV plate is flipped so that the drainage port faces the scleral surface, while the tube is inserted via the pars plana [5]. This modification was designed to minimize anterior segment-related complications and to optimize bleb morphology. The present study builds upon our initial report by evaluating long-term clinical outcomes, postoperative complications, imaging findings, including orbital magnetic resonance imaging (MRI) assessment of bleb morphology [6], and corneal endothelial cell density (ECD) changes associated with this technique in the same cohort with extended follow-up. Accordingly, the primary objective of this extended follow-up analysis was to evaluate long-term intraocular pressure (IOP) control after upside-down pars plana AGV implantation. Secondary objectives were to assess glaucoma medication burden, postoperative complications and reinterventions, long-term bleb morphology on orbital MRI (in available cases), and changes in corneal ECD.

## Materials and methods

Study design and patients

This retrospective observational study represents a long-term follow-up analysis of the same cohort previously reported in our original description of the upside-down pars plana AGV implantation technique [5]. The surgical approach, inclusion/exclusion criteria, and baseline characteristics were identical to those of the initial report. In the present extended analysis, we updated long-term clinical outcomes, postoperative complications, imaging findings of bleb morphology, and corneal ECD over a longer observation period.

This study adhered to the tenets of the Declaration of Helsinki and was approved by the Institutional Review Board of the Jikei University School of Medicine (no. 30-404(9425)). The requirement for written informed consent was waived, and all data were deidentified prior to analysis.

Surgical technique

The surgical procedure was identical to that described in our original publication [5]. All procedures were performed by the same experienced glaucoma surgeon (YM). Following a standard pars plana vitrectomy, an AGV (Model FP7; New World Medical, Rancho Cucamonga, CA) was implanted with the plate flipped (“upside-down”) so that the drainage port faced the scleral surface [5]. The valve plate was positioned 8-10 mm posterior to the limbus, and the tube was inserted into the vitreous cavity after vitrectomy through a pars plana sclerotomy. Adjunctive mitomycin C (MMC) was not used in any case.

Outcome measures

The primary outcome was IOP during long-term follow-up. Secondary outcomes included glaucoma medication score, postoperative complications, corneal ECD, and bleb morphology assessed by MRI.

The glaucoma medication score was calculated as previously described [5]: 1 point for each single-agent topical medication, 2 points for each fixed-combination topical medication, and 2 points for oral carbonic anhydrase inhibitor use.

Postoperatively, patients were managed with a standardized regimen of topical antibiotics and topical corticosteroids for four weeks. Follow-up visits were scheduled at postoperative day 1, week 1, month 1, and then every one to three months thereafter, with glaucoma medications adjusted at each visit based on IOP and clinical findings.

Magnetic Resonance Imaging

Orbital MRI was performed in two eyes at approximately six years postoperatively using a 1.5-T scanner (Magnetom Avanto; Siemens, Erlangen, Germany). The imaging protocol was identical to that used in our initial report [5] and included 3D true Fast Imaging with Steady-state Precession (repetition time (TR)/echo time (TE) 5.24/2.28 ms; slice thickness 0.6 mm) and 3D Constructive Interference in Steady State (TR/TE 10.74/5.37 ms; slice thickness 0.8 mm). Coronal and axial images were evaluated for peri-endplate bleb morphology, including a double-layered fluid configuration.

Corneal Endothelial Cell Assessment

Corneal ECD was measured preoperatively and postoperatively using specular microscopy (Konan Specular Microscope X V; Konan Medical, Japan). To account for differences in follow-up duration, the average monthly rate of ECD loss was calculated by dividing the total reduction in ECD by the number of months of observation.

Statistical analysis

Statistical analyses were performed using IBM Statistical Package for the Social Sciences Statistics (version 22.0; IBM Corp., Armonk, NY) under a valid license. Continuous variables were compared using paired t-tests, and p < 0.05 was considered statistically significant. Kaplan-Meier survival analysis was used to assess long-term surgical success.

Surgical failure was defined as any of the following: 1) IOP >21 mmHg despite maximally tolerated medical therapy, 2) IOP <5 mmHg on two consecutive visits after postoperative month 1, or 3) reoperation for glaucoma. Eyes meeting any of these criteria were classified as Category A failures; those with concurrent loss of vision during follow-up were classified as Category B failures. Surgical success was defined as the absence of failure.

## Results

Patient characteristics

A total of 10 eyes from 10 patients (eight male and two female patients) were included. The mean age was 57.8 ± 12.1 years. Glaucoma types included neovascular glaucoma (NVG; n = 7) and one case each of primary open-angle glaucoma, iridocorneal endothelial syndrome, and postoperative total peripheral anterior synechiae following proliferative diabetic retinopathy. The mean follow-up duration was 45.9 ± 24.2 months (Table 1).

**Table 1:** Baseline characteristics and surgical details of eyes undergoing upside-down pars plana AGV implantation IOP: intraocular pressure; AGV: Ahmed glaucoma valve

Variable	Value
Number of eyes (patients)	10 (10)
Age (years), mean ± SD	57.8 ± 12.1
Sex	
Male	8
Female	2
Diagnosis	
Neovascular glaucoma	7
Primary open-angle glaucoma	1
Iridocorneal endothelial syndrome	1
Total peripheral anterior synechiae after proliferative diabetic retinopathy	1
Previous glaucoma surgery	
Trabeculectomy	4
Glaucoma drainage device surgery	1
Cyclocryotherapy	1
Previous vitrectomy	7
Baseline IOP (mmHg), mean ± SE (range)	35.8 ± 4.4 (19-65)
Preoperative glaucoma medication score, mean ± SE (range)	4.6 ± 0.7 (0-7)
Follow-up duration (months), mean ± SD; median (range)	45.9 ± 24.2; 49.5 (11-76)
AGV plate location	
Superotemporal	7
Superonasal	2
Inferotemporal	1

IOP and medication burden

**Figure 1:**
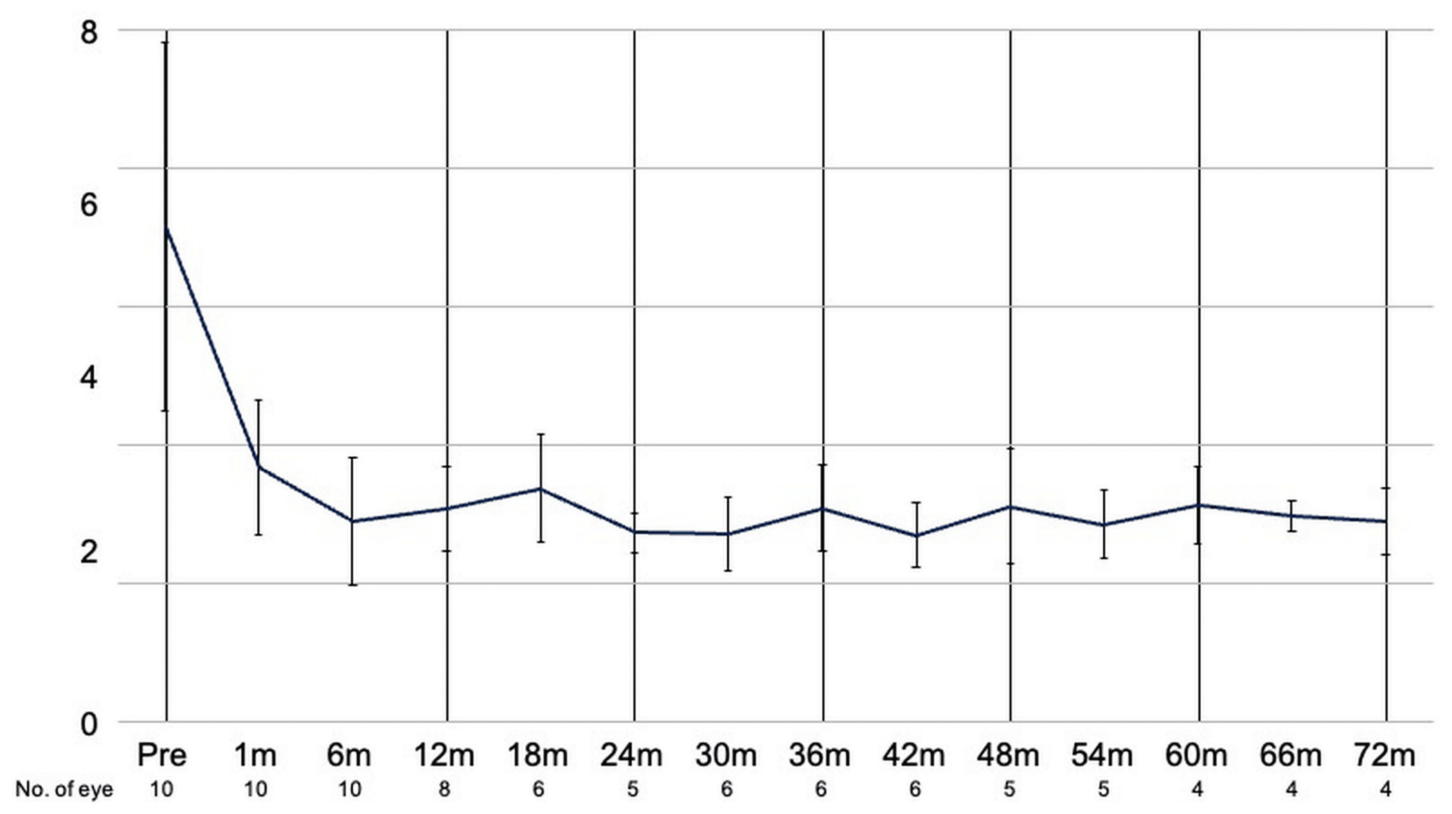
IOP over time after upside-down pars plana Ahmed glaucoma valve implantation Mean IOP at baseline and at each postoperative follow-up time point. Error bars indicate ±1 standard error of the mean. Sample size at each time point is shown in the X-axis IOP: intraocular pressure

Mean IOP decreased from a preoperative value of 35.8 ± 4.4 to 15.4 ± 1.1 mmHg (n = 8) at one year, 15.5 ± 1.3 mmHg (n = 6) at three years, 15.7 ± 1.4 mmHg (n = 4) at five years, 14.9 ± 0.6 mmHg (n = 4) at 66 months, and 14.5 ± 1.2 mmHg (n = 4) at 72 months postoperatively. Compared with baseline, IOP was significantly lower than preoperative values at all time points from 1 to 54 months and at 66 months postoperatively (p < 0.05, paired t-test; Figure 1). Transient IOP elevation (>21 mmHg) occurred in seven patients but resolved spontaneously or with additional topical medications. No patient required ocular massage, needling, or additional glaucoma surgery during follow-up.

**Figure 2:**
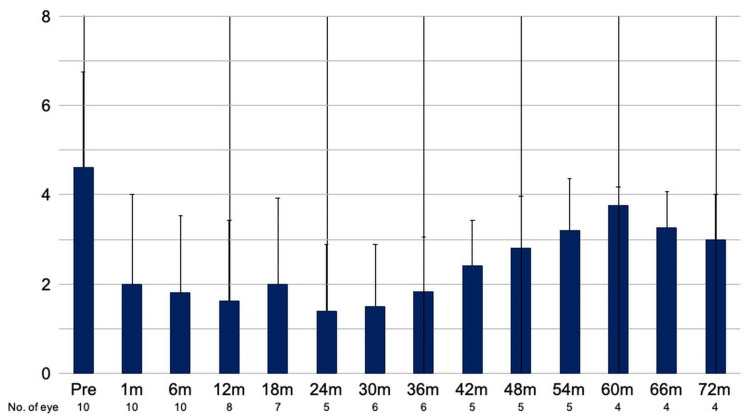
Glaucoma medication score over time after upside-down pars plana Ahmed glaucoma valve implantation Mean glaucoma medication score at baseline and each postoperative follow-up time point (see the Methods section for score definition). Error bars indicate ±1 standard error of the mean. Sample size at each time point is shown in the X-axis

The mean glaucoma medication score decreased from 4.6 ± 0.7 preoperatively to 1.6 ± 0.6 (n = 8) at one year, 1.8 ± 0.5 (n = 6) at three years, 3.8 ± 0.2 (n = 4) at five years, and 3.0 ± 0.5 (n = 4) at 72 months postoperatively. A significant reduction in medication score was observed through 42 months postoperatively (p < 0.05; Figure 2).

Complications

One patient developed transient hypotony at three and four months postoperatively, which resolved without intervention. One eye experienced an intraoperative suprachoroidal hemorrhage and subsequently lost light perception at seven months. Two additional eyes, despite stable IOP, lost light perception at 52 and 54 months postoperatively, which was judged to be attributable to progressive retinal ischemia related to the underlying disease rather than device-related complications of the procedure. No other vision-threatening complications occurred. No cases of diplopia or ocular motility disturbance were observed during follow-up.

Surgical success

**Figure 3:**
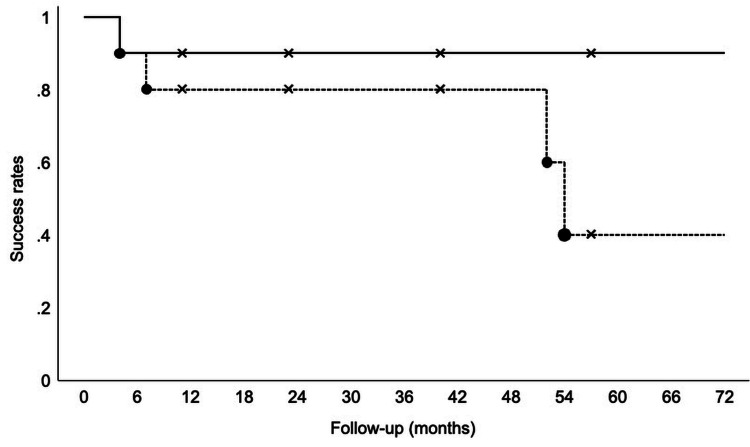
Kaplan-Meier survival analysis of surgical success Kaplan-Meier curves show cumulative success for Category A (meeting failure criteria) and Category B (Category A failure with concurrent loss of vision). Cross marks indicate censored observations. Follow-up is shown in months

Kaplan-Meier survival analysis showed a cumulative success rate of 90% at four months in Category A. In Category B, the cumulative success rate was 80% at seven months, 60% at 52 months, and 40% at 54 months (Figure 3).

MRI findings

**Figure 4:**
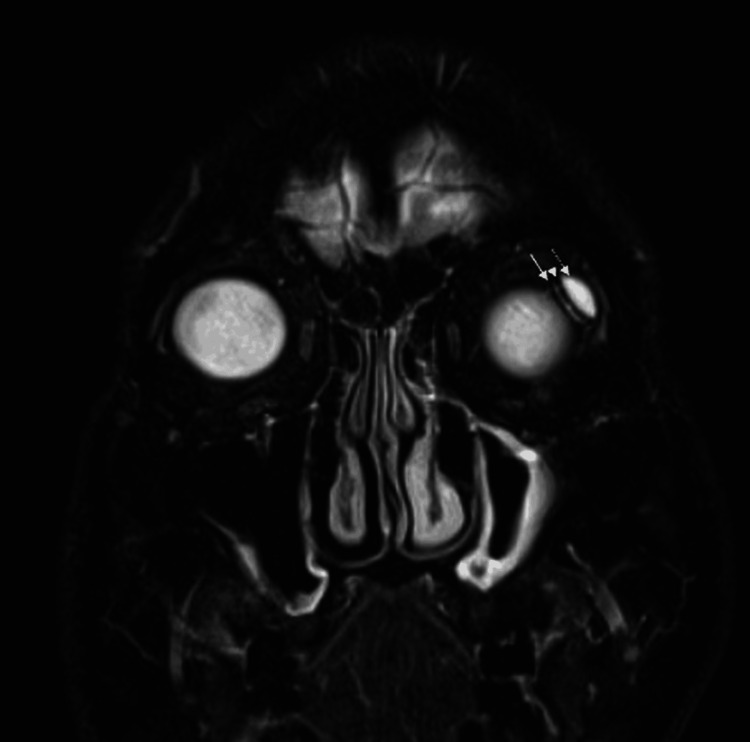
Orbital MRI after upside-down pars plana Ahmed glaucoma valve implantation The operated eye is shown on the right side of the image. A representative T2-weighted coronal orbital MRI obtained at approximately six years postoperatively demonstrates a hyperintense fluid signal consistent with a peri-endplate bleb. A double-layered bleb is observed on the scleral side (solid arrow) and conjunctival side (dashed arrow) of the endplate. The endplate is identified as a low-intensity structure between the bilayered bleb (arrowheads) MRI: magnetic resonance imaging

**Figure 5:**
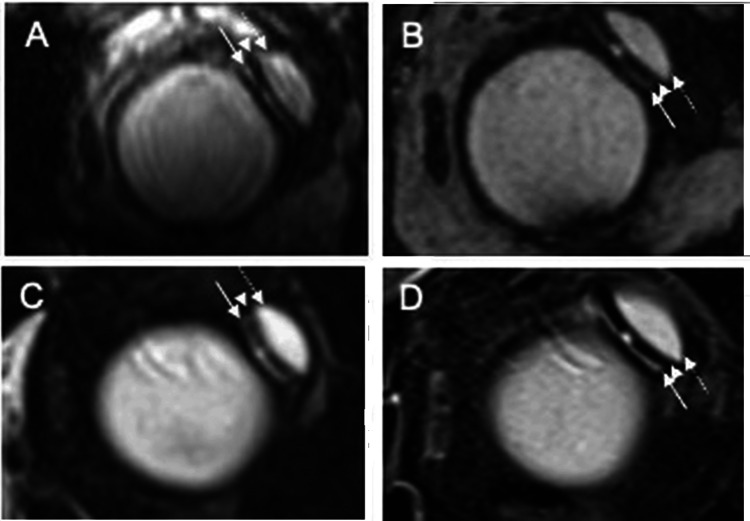
Long-term preservation of double-layered bleb morphology on orbital MRI (A,C) Images from the same operated eye (Eye 1). (B,D) Images from another operated eye (Eye 2). (A,B) T2-weighted images obtained at approximately nine months postoperatively. (C,D) Corresponding images obtained at approximately six years postoperatively. A hyperintense fluid signal consistent with aqueous fluid forms a double-layered bleb on the scleral side (solid arrow) and conjunctival side (dashed arrow) of the endplate. The endplate is identified as a low-intensity structure between the bilayered bleb (arrowheads) MRI: magnetic resonance imaging

As described in our previous report, postoperative MRI demonstrated a hyperintense fluid signal surrounding the AGV endplate, consistent with a double-layered bleb [5]. In the current study, orbital MRI at approximately six years postoperatively confirmed preservation of this double-layered bleb configuration on both the conjunctival and scleral sides of the endplate in two eyes, resembling the findings observed at approximately nine months (Figures 4, 5). Solid arrows indicate the scleral-side fluid compartment, dashed arrows indicate the conjunctival-side compartment, and arrowheads indicate the endplate.

Corneal endothelium

The average monthly rate of corneal endothelial cell loss was 0.28% ± 0.26%.

## Discussion

In this study, we evaluated the long-term efficacy and safety of the upside-down technique for pars plana AGV implantation in refractory glaucoma, with long-term IOP control as the primary endpoint and medication burden, complications/reinterventions, MRI-based bleb morphology (in available cases), and corneal endothelial outcomes as secondary endpoints. Over a follow-up period extending up to six years, this approach demonstrated sustained IOP reduction through 66 months and a favorable safety profile, supporting its potential as a viable surgical option in complex or high-risk cases.

Tube shunt surgery has long been employed in the management of refractory glaucoma, particularly in eyes with a high risk of surgical failure, such as those with NVG [7]. Large randomized trials have established the effectiveness of glaucoma drainage implants in refractory glaucoma [8,9]. However, outcomes in NVG remain suboptimal due to factors such as intraocular inflammation and elevated levels of cytokines and vascular endothelial growth factor (VEGF), which may promote fibrotic encapsulation around the endplate and ultimately lead to bleb failure [3,10]. A pooled analysis by Bowden et al. demonstrated that NVG was significantly associated with tube shunt failure in univariate analysis [4]. Chronic conjunctival inflammation in NVG may also contribute to dense postoperative fibrosis around the implant, increasing outflow resistance and contributing to both early postoperative hypertensive phases and long-term surgical failure.

To overcome these limitations, adjunctive approaches such as MMC and intravitreal anti-VEGF therapies have been investigated [11]. However, MMC remains off-label in many countries, thereby limiting its widespread applicability [12]. Although Kaushik et al. reported that combining AGV implantation with intravitreal anti-VEGF reduced the incidence of postoperative hyphema, this approach did not significantly improve long-term surgical success compared to AGV implantation alone [13]. Consequently, despite these adjunctive strategies, the overall prognosis for NVG remains unsatisfactory.

The upside-down AGV technique offers a novel surgical alternative that may circumvent some of these issues. In our study, sustained IOP reduction was achieved without the use of MMC or anti-VEGF agents. Compared with conventional AGV implantation, in which the five-year postoperative IOP typically declines by approximately 56% [14], our cohort showed a 59.5% reduction at six years, with the mean IOP decreasing from 35.8 to 14.5 mmHg. Although the sample size was small, these results are encouraging. Transient postoperative IOP elevation occurred in seven patients, all of whom were successfully managed with either observation or topical medications, and none required ocular massage, needling, or revision surgery.

One recognized limitation of conventional AGV surgery is the development of a fibrous capsule around the endplate, which can increase resistance to aqueous outflow and contribute to postoperative IOP elevation. A postoperative hypertensive phase is a well-recognized phenomenon after AGV implantation and may be particularly relevant in NVG [15]. In contrast, the transient nature of postoperative IOP elevation observed in our series suggests that the upside-down technique may reduce the incidence or severity of such outflow resistance. With this technique, the drainage port is oriented toward the scleral surface, and the adjacent tissue environment (sclera and Tenon’s capsule) may be less prone to forming a restrictive capsule or exerting compressive resistance along the outflow pathway.

Supporting this hypothesis, orbital MRI obtained at approximately six years postoperatively demonstrated persistence of a double-layered peri-endplate fluid configuration in two eyes (Figures 4, 5), resembling the appearance observed at approximately nine months. Although MRI cannot directly quantify fibrosis, the sustained bilayered fluid distribution suggests stable long-term bleb morphology in these imaged cases. Iwasaki et al. similarly reported that Baerveldt blebs exhibiting a double-layered structure were associated with lower postoperative IOP than those with single-layered blebs [6]. The long-term maintenance of this bleb morphology may, therefore, contribute to durable IOP control without adjunctive antifibrotic agents.

Regarding complications, one patient experienced an intraoperative suprachoroidal hemorrhage and subsequently lost light perception for seven months. Two additional patients lost light perception during long-term follow-up despite well-controlled IOP, likely due to progressive ischemic changes related to ocular ischemic syndrome and proliferative diabetic retinopathy, respectively. No other serious complications were observed. Corneal endothelial cell loss remained minimal and was within an acceptable range compared to previous AGV studies [1,2]. No cases of diplopia or ocular motility disturbances were reported.

This study has several limitations. First, this was a single-center retrospective study with a small sample size, which limits the generalizability of the findings. Second, MRI evaluation of bleb morphology was performed in only two eyes; therefore, the observed long-term double-layered configuration may not represent all cases. Third, the number of eyes available for analysis decreased at later follow-up time points, which may introduce attrition bias. Larger prospective studies are warranted to confirm the long-term efficacy and to better characterize bleb morphology after the upside-down technique.

## Conclusions

In this retrospective cohort with follow-up extending to six years, the upside-down pars plana AGV technique achieved sustained IOP reduction through 66 months and reduced medication burden without the need for ocular massage, needling, or glaucoma reoperation. Long-term imaging in two eyes suggested preservation of a characteristic double-layered peri-endplate bleb configuration. Larger prospective studies are warranted to confirm these findings and to better define the indications for this technique in refractory glaucoma, including NVG.
